# Broadband and high-power terahertz radiation source based on extended interaction klystron

**DOI:** 10.1038/s41598-019-41087-3

**Published:** 2019-03-14

**Authors:** Renjie Li, Cunjun Ruan, Ayesha Kosar Fahad, Chenyu Zhang, Shasha Li

**Affiliations:** School of Electronic and Information Engineering, Beihang University, Beijing, 100191 China

## Abstract

Terahertz applications require high performance and high reliability terahertz radiation sources, especially the urgent demands of high output power and broad bandwidth. The extended interaction klystron (EIK) has the great potential to generate hundreds of watt output power in terahertz band. The terahertz EIK adopts multiple gap cavities and unequal-width slots structure is proposed with methodological improvement of bandwidth and output power. The unequal-width slots are the key design of the multiple gap cavity, and the influences of unequal-width slots on the electromagnetic field distribution and beam-wave interaction are analyzed in detail. With multiple gap cavities and unequal-width slots structure, EIK has advantages of wider frequency separation and larger effective characteristic impedance. Particle in cell (PIC) simulation indicates that the bandwidth of unequal-width slots structure can reach to 550 MHz in our initial G-band EIK design. Then, we utilize two kinds of resonance cavities with different width ratios to build a six-cavity beam-wave interaction system and make it operate at the state of stagger-tuning, the bandwidth can be extended to 1–1.5 GHz. Our research shows that the unequal-width slots structure has wider tuning frequency range. Furthermore, the bandwidth can be further broadened to over 2 GHz when dynamic-tuning is adopted, while maintains a high output power of 560 W with efficiency of 11.3% and gain of 47.5 dB. Thus, the methods of multiple gap cavities with unequal-width slots structure, stagger-tuning and dynamic-tuning are much important for the bandwidth improvement of EIK in terahertz band.

## Introduction

The wide application prospects of millimeter-wave (MMW) and terahertz (THz) technology, such as atmospheric sensing, high-resolution imaging, satellite communications, active denial and next generation acceleration drivers^1–3^, has presented fresh impetus to the growth of vacuum electronics devices (VEDs)^4–7^. However, VEDs will be faced many challenges when the frequencies are explored into MMW and THz range, such as assembly of the miniature circuit with high precision and large output power as well as broad bandwidth. Extended interaction klystron (EIK) is a promising VED to solve those problems and have proven to be compact and reliable THz sources^8,9^, which was proposed by Chodorow and Wessel-Berg in 1960s^10^. EIK is made up of multi-gap cavities with planar-feature ladder circuit, which is simple in geometry and compatible to the available microfabrication technologies^11,12^. Moreover, the multi-gap ladder circuit increases the characteristic impedance (*R/Q*) and enables a high gain per circuit length. The shorter circuit length would alleviate the beam interception for a fine tunnel and make it more suitable for permanent magnetic focusing, thus to increase the operation stability and long-life of the whole system. EIK is a hybrid of travelling wave tube (TWT) and klystron, the structure will make EIK possess great potentials at output power, efficiency, and bandwidth in MMW and THz applications^4,13^.

EIK has been developed for several decades until now, extraordinary performances of W-band EIKs have been successfully demonstrated^13,14^, while the EIK concept has proved highly scalable at frequencies less than 220 GHz and the modern EIK technology optimizes performance up to 280 GHz^15^. CPI Canada has been producing EIKs across a broad range of frequencies. At 95 GHz, CPI has established a pulsed-power performance of 2 kW peak for 300 MHz bandwidth^13^. A novel development of coupling to more than one cavity resonance has been undertaken, this manner can significantly increase the EIK bandwidth while without a proportional loss of peak efficiency^16^. Furthermore, G-band EIK with bandwidth of 300 MHz and output power of 7 W has been produced^17^, and G-band sheet beam EIK with 453 W power and 41.6 dB achieved in Naval Research Laboratory of America has been fabricated^18^.

In our previous works, we have perfected the design methodology and discussed the approaches to improve the output power of EIK, obtained a simulation bandwidth of about 500 MHz^19,20^. In EIK, except the demand of high output power, the wide bandwidth is also important in the MMW and THz applications. The operation bandwidth of current EIK needs to be further increased. Thus, we emphatically discuss the methods to extend the bandwidth, including the unequal-width slots structure, stagger-tuning technology and dynamic-tuning technology. The simulation results indicate that the final bandwidth can reach to 2 GHz, which is considerable wide for the G-band EIK.

## Electromagnetic Field Distribution and High-frequency Properties

**Figure 1:**
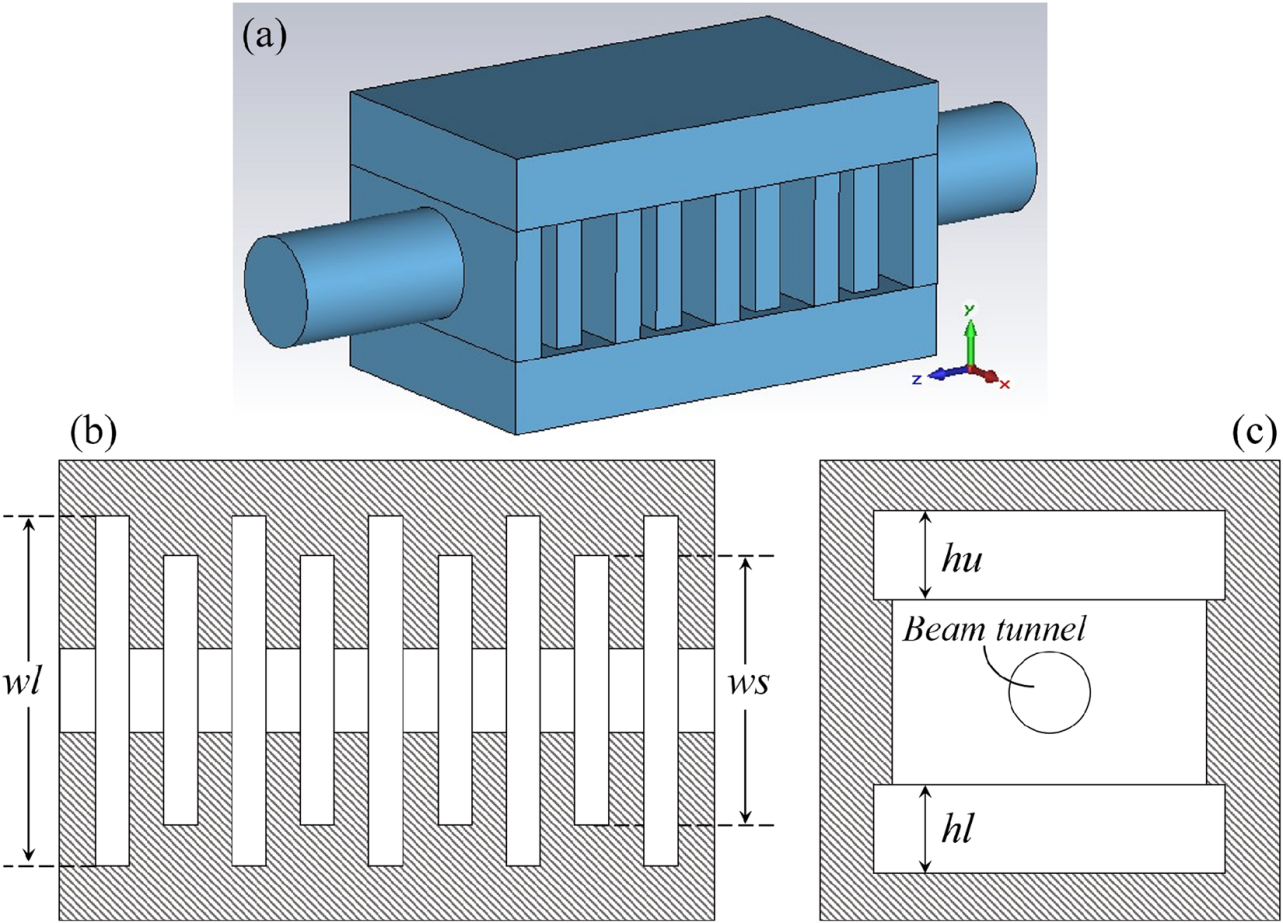
Schematic diagram of unequal-width slots resonance cavity. (**a**) 3D model. (**b**) *x*-*z* cross section. (**c**) *x*-*y* cross section.

The schematic diagram of unequal-width slots structure with nine gaps is shown in Fig. 1. Figure 1(a) is the 3D model including circular electron beam tunnel, coupled cavities, and periodic long-slots and short-slots. Figure 1(b) is the *x*-*z* cross section view, with five long-slots and four short-slots interleaving along axial direction (*z*-axis). The width of long-slot and short-slot is denoted as *wl* and *ws*, respectively. The values of *wl* and *ws* not only affect the frequency distribution, but also affect the high-frequency properties. For research convenience, we define the ratio of *wl* with *ws* as α. Figure 1(c) is the view of *x*-*y* cross section, where *hu* and *hl* denote the height of lower and upper coupled cavities. We select TE_10_-π mode as the operating mode, which has the same electric fields in alternate gaps and mutually reverse fields in adjacent gaps.

### Optimization of *α* on electric field distribution

**Figure 2:**
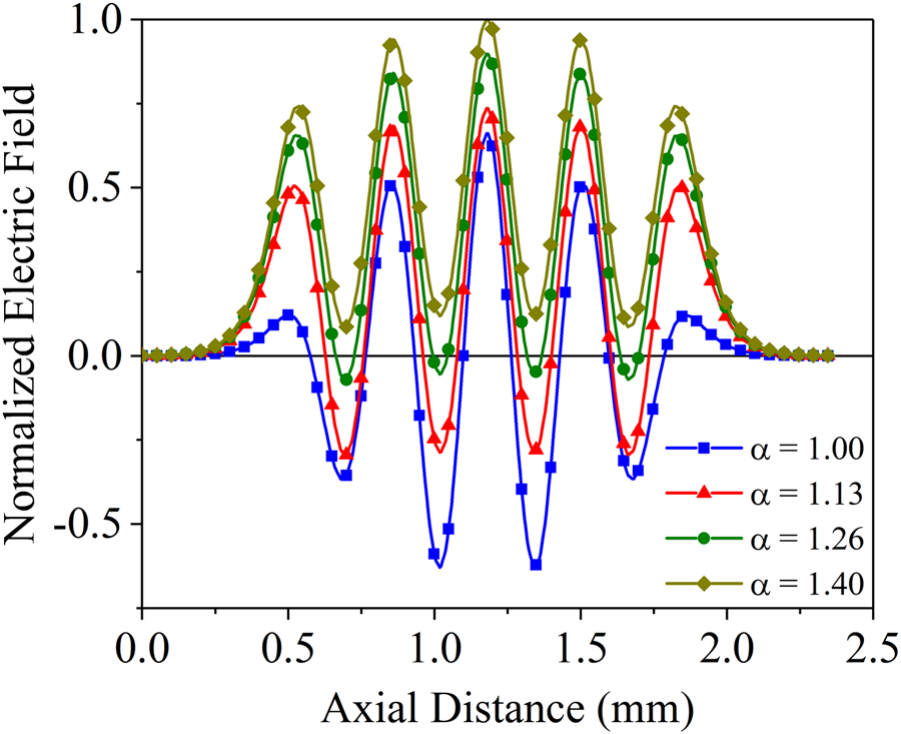
Normalized axial electric field distribution of π mode under different *α*.

The resonance properties are related with the structure shapes and parameters, especially the dimensions of long-slots and short-slots. Due to the effective beam-wave interaction mainly occurs between the electron beam with the longitudinal electromagnetic wave, thus we only concern the longitudinal electromagnetic wave. For the structure shown in Fig. 1(a), the longitudinal electromagnetic wave only exists the electric field while the magnetic field is zero. The normalized axial electric field distribution of π mode in tunnel center is shown in Fig. 2. As *α* increases, the field curves present the trend of shifting up. As *α* = 1.00 corresponding to the identical slots structure, the field intensity in both sides of the cavity are much weaker than the center. As *α* = 1.26, the field of short-slots are almost zero, implying no beam-wave interaction in short-slots. As *α* increases to 1.40, the fields of short-slots and long-slots are in same phase, which deviates from the electric field distribution of π mode. Supposing the electric field of long-slots and short-slots are positive at time *t*_0_, the electrons are firstly accelerated by long-slot, then the electric field of long-slots and short-slots will become negative at *t*_1_ after a period, the accelerated electrons will enter into the adjacent short-slot and will be decelerated. Thus, the electric field of short-slots may generate negative effect for beam modulation as *α* = 1.40. As *α* = 1.13, the field intensity of long-slots or short-slots are both comparatively uniform, and the field distribution is in coincidence with π mode, which may generate positive beam-wave interaction in all gaps.

**Figure 3:**
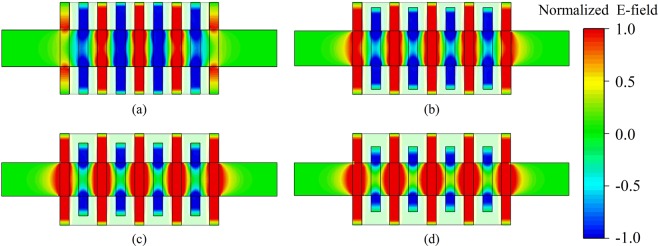
Electric field patterns of π mode under different *α*. (**a**) *α* = 1.00. (**b**) *α* = 1.13. (**c**) *α* = 1.26. (**d**) *α* = 1.40.

The electric field patterns of π mode are illustrated in Fig. 3, and they are respectively corresponding to the four kinds of *α* in Fig. 2. With the increase of *α*, the width difference of long-slots with short-slots become larger, which makes the field isolation between the long-slots and short-slots worse. For *α* = 1.26 and *α* = 1.40, we can clearly see that the fields in short-slots become weaker while the fields in long-slots become stronger. The strong positive fields in long-slots will leak into short-slots, making the negative fields of short-slots become positive. For the structure of *α* = 1.13, it has a very good field isolation, and the field in each long-slot or short-slot is strong and uniform. Such electric filed distribution will be highly conducive to the beam-wave interaction.

### Optimization of *α* on high-frequency properties

**Figure 4:**
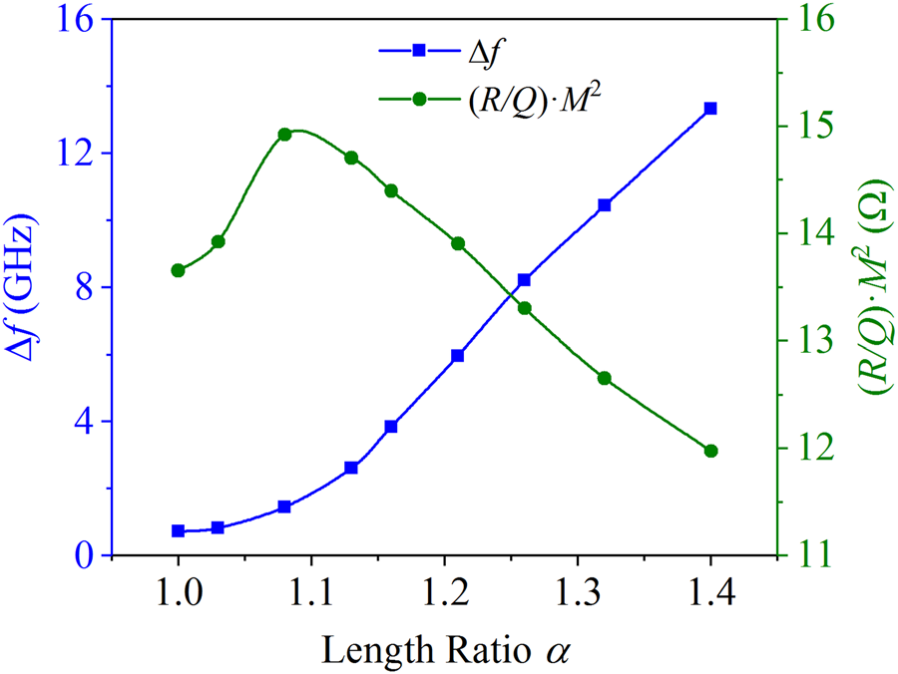
The relationship of *α* with Δ*f* and (*R/Q*)**·***M*^2^.

The influence of *α* on frequency separation Δ*f* and effective characteristic impedance (*R/Q*)**·***M*^2^ is shown in Fig. 4. With the increase of *α*, Δ*f* significantly increases. On one hand, the larger Δ*f* makes the unequal-width slots structure more easily avoid mode competition than the identical slots structure. On the other hand, the unequal-width slots structure can adopt more gaps compared with the identical slots structure under the same Δ*f*, which is beneficial for increasing *R/Q*. That is the reason we adopt unequal-width slots structure in the design of G-band EIK. With the increase of *α*, (*R/Q*)**·***M*^2^ firstly increases and then decreases and reaches to maximum at *α* = 1.08. Usually, the larger (*R/Q*)**·***M*^2^ will generate stronger beam-wave interaction, acquiring higher gain-bandwidth product. However, we should consider the power and stability synthetically. A very narrow Δ*f* will easily cause mode competition, leading to the operation instability of the whole system.

## Beam-Wave Interaction Analysis

To verify the influence of *α* on the beam-wave interaction properties, we constructed a four-cavity EIK in CST^21^ to conduct PIC simulation, with the assumption of 0.30 A beam current and 16.5 kV voltage. It should be noted that the design procedure and physical model of the four-cavity EIK can be referred to our previous work^17^.

### Optimization of *α* on output performance

**Figure 5:**
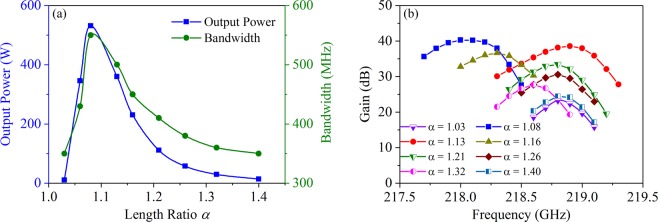
(**a**) The influence of *α* on output power and bandwidth. (**b**) The relationship of gain with frequency under different *α*.

With the four-cavity scheme, Fig. 5(a) shows the influence of *α* on output power and bandwidth, which shows the same trends of firstly increasing and then decreasing and both reach to maximum at *α* = 1.08. The output power decreases rapidly at both sides of *α* = 1.08, and there is almost no power output at *α* = 1.03 and 1.40 compared with the high output power of 530 W at *α* = 1.08. Furthermore, the bandwidth also increases to 550 MHz at *α* = 1.08 while it is only 350 MHz at *α* = 1.03 and 1.40. Comparing Fig. 5(a) with Fig. 4, it should be noted that the variation trends of output power and bandwidth are in agreement with (*R/Q*)**·***M*^2^. Thus, by optimizing the widths of long-slots and short-slots, we can get the maximum (*R/Q*)**·***M*^2^, which will lead to the higher output power and wider bandwidth. However, the frequency separation should be also considered when we select the widths of long-slots and short-slots. For the designed bandwidth, the minimum frequency separation between the operating mode and the adjacent modes should be greater than the half of bandwidth. From Fig. 4, we noticed that the frequency separation is over 1.00 GHz when *α* ≥ 1.08, which is wide enough to avoid mode competition in terms of the 1–2 GHz bandwidth requirement.

Figure 5(b) plots the relationship of gain with frequency under different *α*. It should be noted that the resonance frequency is mainly co-determined by the widths of long-slot and short-slot, our designed center frequency is around 218.0–219.0 GHz. However, considering the practical fabrication precision while keeping other parameters unchanged, we are unable to adjust the widths of long-slot and short-slots limitlessly to resonate at the same frequency under different *α*. Therefore, the slight differences of frequency shown in Fig. 5(b) are acceptable. We can clearly find that the gains of *α* = 1.08 and *α* = 1.13 are much larger than the other cases of *α*. On the other hand, we also noticed there is an overlap between the high-frequency band of *α* = 1.08 and the low-frequency band of *α* = 1.13. Therefore, we may simultaneously utilize the two cases of *α* to further extend the bandwidth. The basic idea is that we can design a beam-wave interaction system including the two types of resonance cavities, and make the operation frequency fall within the bandwidth ranges determined by *α* = 1.08 and *α* = 1.13.

**Figure 6:**
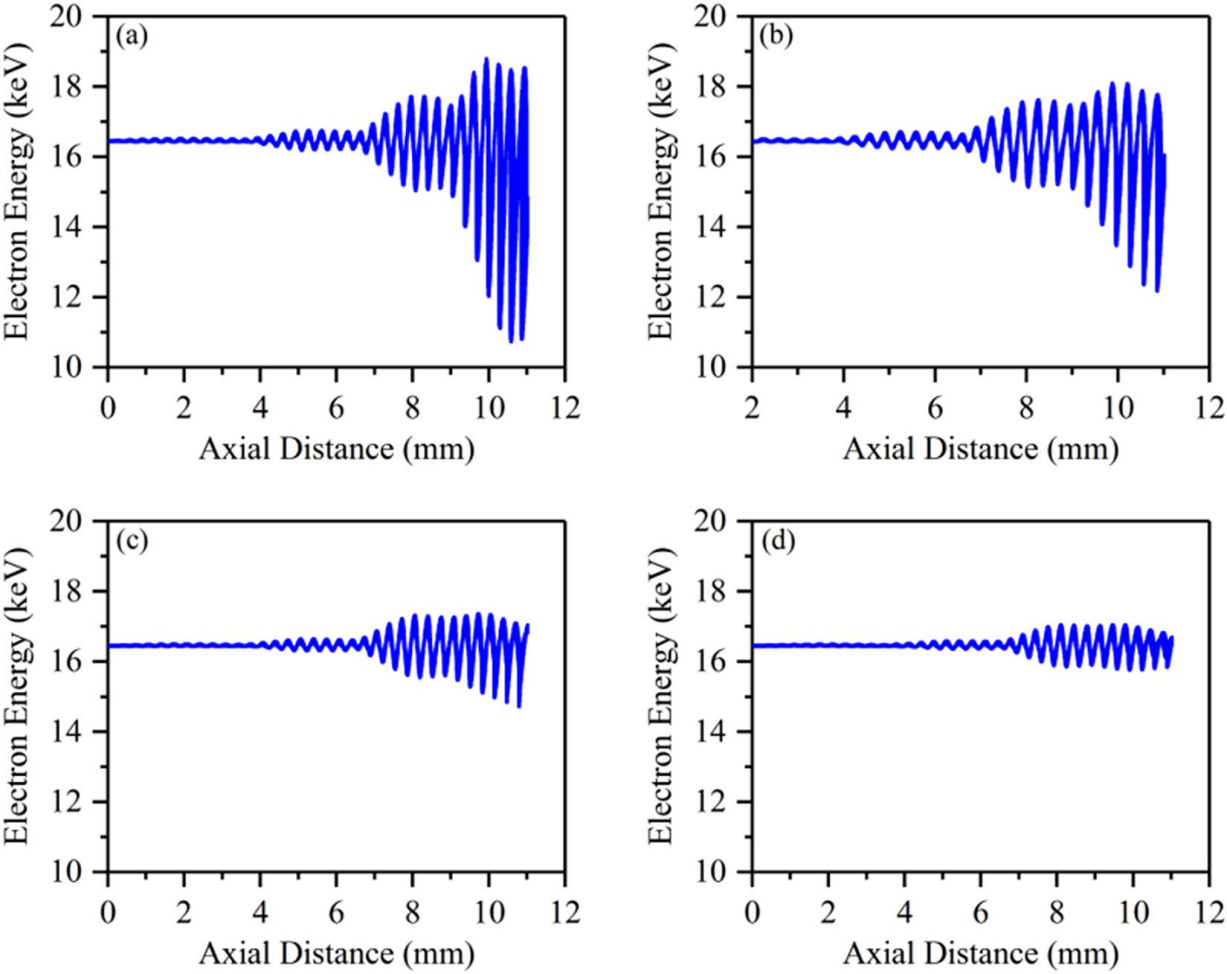
The particles phase-spaces of electron beam under different *α*. (**a**) *α* = 1.08. (**b**) *α* = 1.13. (**c**) *α* = 1.26. (**d**) *α* = 1.40.

The influence of *α* on the beam-wave interaction can be clearly seen from the particles phase-spaces, as shown in Fig. 6. For *α* = 1.08, the effect of acceleration and deceleration of the electron beam is significant, most electrons are decelerated in output cavity and the lowest energy is about 11 keV. For *α* = 1.13, the effect of energy conversion is also relative significant. However, for *α* = 1.26 and 1.40, the effect of acceleration and deceleration becomes inapparent and the energies of most electrons maintain around 16.5 keV, which indicates that the beam-wave interactions are very weak under the two cases.

**Figure 7:**
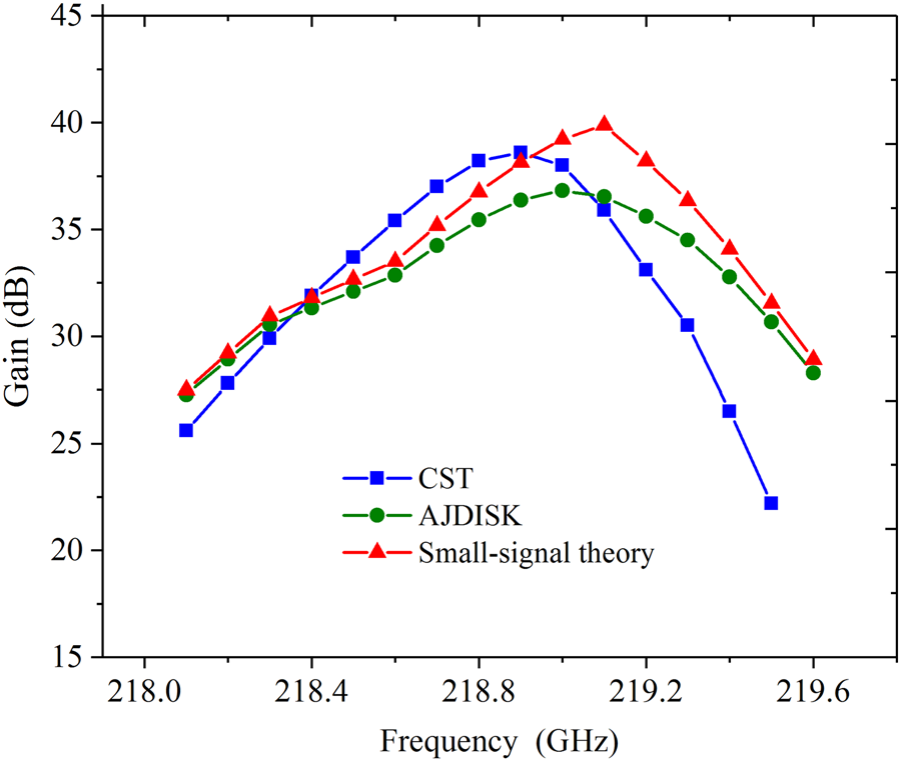
Gain versus the frequency of the small-signal theory, AJDISK and CST.

To verify the reliability of CST simulations, we compared the PIC simulations with the small-signal theory analysis and 1D AJDISK^22,23^ calculations. We take *α* = 1.13 for example and the compared results are shown in Fig. 7. The corresponding frequency of the maximum gain in the small-signal theory and AJDISK is a little higher than that of CST, which might be due to the beam loading effect in CST PIC simulation, as well as the approximations in the small-signal theory and AJDISK. However, it should be noted that the small-signal theory, AJDISK and CST have the similar variation tendencies with the maximum gains of 40.1 dB, 36.8 dB and 38.6 dB, and −3dB bandwidths of 480 MHz, 600 MHz and 500 MHz, respectively. Thus, the approximate results from the three curves indicate that the CST simulations are reliable and valid.

### Stagger-tuning

**Figure 8:**
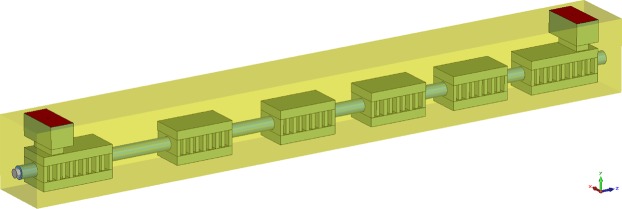
3D beam-wave interaction simulation model with six-cavity constructed in CST.

We redesigned a six-cavity beam-wave interaction system, which involves the cavities of *α* = 1.08 and *α* = 1.13. The 3D simulation model is illustrated in Fig. 8, and the cavity material is set as oxygen-free copper with effective conductivity of 2.36 × 10^6^ S/m. The resonance frequency and characteristic parameters of each cavity are listed in Table 1. The effective characteristic impedance of each cavity is very close, to ensure the strong beam-wave interaction in each cavity. Meanwhile, the input cavity and output cavity resonate at the center frequency, while the middle cavities resonate on both sides of the center frequency. Such design will make the beam-wave interaction system operate at the state of stagger-tuning, which is in great possibility to extend the bandwidth. The ideal uniform magnetic field of 0.75 T is applied to confine and focus the electron beam in tunnel with the total length of 1.54 cm.

**Table 1:** Characteristic parameters of each cavity.

*N*	*f* (GHz)	*R/Q* (Ω)	*M*	(*R/Q*)·*M*^2^ (Ω)	*Q*_0_	*Q*_*e*_
1	220.21	45	0.59	15.7	262	101
2	219.58	35	0.65	14.8	257	
3	219.89	35	0.66	15.2	257	
4	220.48	38	0.62	14.6	262	
5	220.77	38	0.62	14.6	262	
6	220.21	45	0.59	15.7	262	101

**Figure 9:**
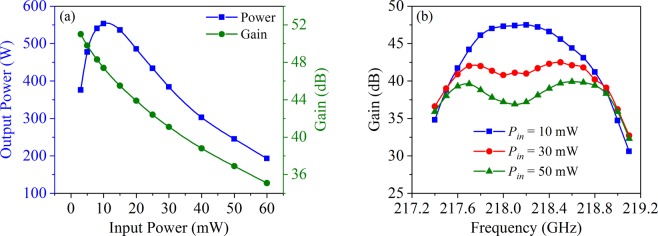
(**a**) The relationship of output power and gain with input power. (**b**) The relationship of gain with frequency under different input power.

The influence of input power on output performance is shown in Fig. 9(a), where we can find that with the increase of input power, the gain gradually decreases while the output power firstly increases and then decreases and reaches to the saturation as input power is 10 mW. Furthermore, we studied the gain-frequency properties under different input power, as illustrated in Fig. 9(b). Comparing the three curves, we can find that there appears gain decline in the center frequency band for input power of 30 mW and 50 mW. The reason for this case is due to the gain oversaturation in the center frequencies for input power of 30 mW and 50 mW. The maximum output power for the three cases of input power are 560 W, 530 W, and 480 W, respectively, and the corresponding bandwidths are 1.0 GHz, 1.3 GHz, and 1.5 GHz, respectively. From the simulation results, due to the adoption of unequal-width slots structure and stagger-tuning technology, the designed beam-wave interaction system has great potential to operate at wide bandwidth of 1.0–1.5 GHz. It should be noted that the frequency shown in Table 1 is the eigen-frequency without the electron beam, as the electron beam is loaded into the whole beam-wave interaction system, the frequency of beam-loaded will decrease to some extent.

### Dynamic-tuning

**Figure 10:**
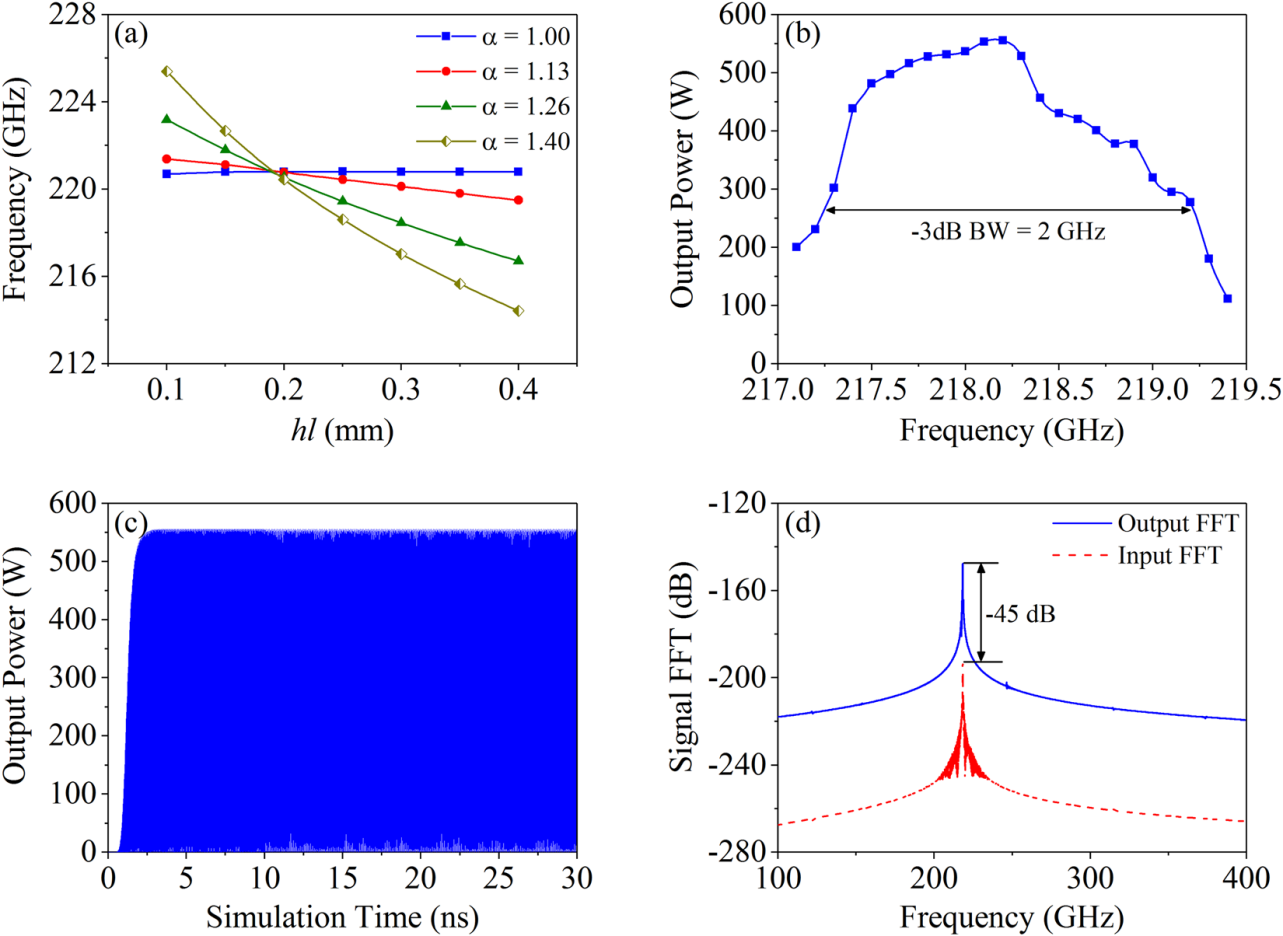
(**a**) The influence of *hl* on frequency under different *α*. (**b**) The relationship of output power with frequency under dynamic-tuning. (**c**) The evolution of output power with time. (**d**) The frequency spectrums of input signal and output signal.

We can adopt dynamic-tuning technology to further extend the bandwidth. By incorporating tuning plunger in each coupled cavity, we can adjust the resonance frequency through changing the height (*hl*) of coupled cavity. This kind of mechanical tuning method can compensate for the frequency shift caused by the fabrication error, also can increase the operation bandwidth. The influence of *hl* on the resonance frequency is shown in Fig. 10(a). For the identical slots structure, the tuning range of frequency is very narrow when *hl* changes. With the increase of *α*, the tuning range gradually increases with the same variation of *hl*. This implies that the unequal-width slots structure has larger capability of frequency tuning, which will be a great superiority in the bandwidth extension compared with the identical slots structure. As far as the output power and dynamic-tuning bandwidth, *α* should be in reasonable range. We noticed that when *hl* varies from 0.10 mm to 0.40 mm, the frequency changes by 2 GHz as *α* = 1.13, which is large enough to tune the frequency in relatively wide range. Thus, in terms of the above six-cavity beam-wave interaction system, the bandwidth can be further extended through dynamic-tuning while maintain the output power at a high level. With the input power of 10 mW, we simulated the relationship of output power with frequency under dynamic-tuning condition, as shown in Fig. 10(b), the mechanical bandwidth reaches to 2 GHz. It can be speculated that the bandwidth has potential to extend to 2.5 GHz or wider if the beam-wave interaction system operates at unequal excitation power. Figure 10(c) shows the evolution of output signal of the center frequency with time, the signal keeps stable after 3 ns and no oscillation was observed. Figure 10(d) illustrates the frequency spectrums of input signal and output signal. We can see that the output signal is very pure with only a single peak in a wide passband, which indicates that the mode competition is well suppressed. Meanwhile, the large difference of 45 dB between the output spectrum and input spectrum means the input signal is effectively amplified through our designed beam-wave interaction system.

## Conclusion

In this paper, we conducted the methodological investigation on bandwidth extension of G-band EIK. The width ratio of long-slot with short-slot has great influence on output power and bandwidth. Under the wide enough frequency separation, the output power and bandwidth increase with the effective characteristic impedance. Utilizing the two kinds of unequal-width structures with different ratios to build a six-cavity beam-wave interaction system, and making each cavity resonate at different frequency, this will extend the bandwidth to 1.0–1.5 GHz. Furthermore, by changing the frequency of each cavity dynamically will further broaden the bandwidth to 2.0 GHz. We can speculate that the bandwidth can be extended to over 2.5 GHz if the system operates at unequal excitation power. EIK as a hybrid of travelling wave tube (TWT) and klystron, who combines the superiorities of wide bandwidth of TWT and high output power of klystron. The relative bandwidth of EIK could reach to 1–2%, but the current bandwidth of practical EIK is still narrow far from the expected performance. The operation mechanism of EIK operating at broadband is still needed to be deeply excavated, including new slow-wave structure, combination of stagger-tuning and dynamic-tuning as well as the overlap of multi-mode. The methods proposed in this paper not only increase the bandwidth of EIK effectively, but also maintain a high level of output power simultaneously. The methodological investigation on bandwidth extension is universal, which can be applied to higher terahertz band. In our current works, we mainly focus on the physical mechanism of EIK with high output power and broad bandwidth, the engineering fabrication will be conducted in the future.
